# Comment on: “The Utility of Thirst as a Measure of Hydration Status Following Exercise-Induced Dehydration”

**DOI:** 10.3390/nu12010215

**Published:** 2020-01-14

**Authors:** Eric D.B. Goulet

**Affiliations:** 1 Faculty of Physical Activity Sciences, University of Sherbrooke, 2500 boul. de l’Université, Sherbrooke, QC J1K 2R1, Canada; 2 Research Centre on Aging, University of Sherbrooke, 1036 Rue Belvédère S, Sherbrooke, QC J1H 4C4, Canada; 3 Hydration and Thermoregulation Laboratory, University of Sherbrooke, 2500 boul. de l’Université, Sherbrooke, QC J1K 2R1, Canada

## Dear Editor-in-Chief

In their study, Adams et al. [1] attempted to determine whether thirst perception could serve as a reliable marker of hydration status during 3 h of exercise where participants either dehydrated by 3% of their body mass or maintained euhydration through water intake, and following exercise where they either could not drink or were allowed to consume water ad libitum during the first 10 min of a 60 min long recovery period. Thus, four conditions were compared: euhydration (fluid intake) during exercise with (1) or without (2) the possibility of replacing (ad libitum) fluid following exercise, and hypohydration (no fluid intake) during exercise with (3) or without (4) the possibility of replacing (ad libitum) fluid following exercise. During exercise, thirst perception adequately tracked fluid status under all conditions, an important observation which, unfortunately, was left mostly undiscussed by the authors. Following exercise, thirst adequately monitored fluid status in 3 out of 4 conditions. However, when participants were not allowed to consume fluid during exercise but could drink ad libitum in the immediate 10 min following exercise, their thirst perception became totally suppressed and satiated over the next 50 min, despite the fact that they had only replaced 55% of their fluid loss within the 10 min drinking period, and by the end of the recovery period they were still hypohydrated by 2.1% of their body mass. This observation led the authors to conclude that “These results represent a limitation in the utility of thirst in guiding hydration practices.” I defend the ideas (1) that their conclusion is precipitated; (2) that the research design used by this study is ecologically invalid and, therefore, that the authors’ conclusion, within the context of their study where no exercise followed the recovery period, and within the hypothetical scenario they proposed, is incorrect and cannot be inferred to real-world exercise conditions; (3) that the alternative solution they present could lead to health and performance issues and is not viable; and (4) that ad libitum fluid replacement may represent the best option under this study scenario and the one contextualized into a real-world context by the authors. As such, I feel that scientists, dietitians, physicians, and physical trainers who will read or have already read this paper deserve to be presented with this alternative point of view. 

I know of no exercise scenario where athletes would be denied fluid intake during a 3 h exercise period performed under harsh conditions and then would only be allowed 10 min for rehydration, with another 50 min of rest where they would again be denied fluid before undergoing a new exercise period. A reasonable and externally valid answer to that research question could have been obtained if in one condition participants had been allowed to drink ad libitum during exercise and throughout the recovery phase following exercise. 

It was unremarkable and to be expected that thirst would become completely suppressed immediately following fluid intake despite no observable changes in blood volume or tonicity [2]. It was, nevertheless, impressive that participants replaced more than 1 L of fluid within the first 10 min following exercise. This behavior may have been physiologically driven by the integration of signals from the oral cavity and the composition of the blood by the subfornical organ, which has been demonstrated to predict how ongoing water consumption will alter fluid balance in the future so as to be able to adjust drinking behavior accordingly [3]. Unfortunately, the authors did not allow participants to continue drinking over the last 50 min of the recovery period. Had they been allowed to do that, additional fluid would have been consumed despite the satiation of thirst [4,5,6,7,8], which would have allowed a greater improvement of fluid balance and enabled the authors to better circumscribe the impact of ad libitum drinking under this scenario. And this is not irrelevant within the context that plasma osmolality had not been restored by the end of the recovery period. Indeed, it has been shown that while thirst is quenched, the activation of the anterior cingulate region of the brain disappears, whereas that of the anterior wall of the third ventricle remains activated, implying that the control of thirst is being performed at the conscious and unconscious levels, the latter being under the regulation of the plasma sodium concentration [9], a reflection of plasma osmolality. 

It is admitted that the feeling of thirst alone would not have provided the impetus to completely restore fluid balance following the recovery period. But the question that should be asked here is: “was it necessary within the context of the current study where there was no period of exercise thereafter?” The answer is no, in that following the recovery period, heart rate, core temperature, urine specific gravity, and thirst pleasantness had all returned to baseline levels despite the persistence of hypohydration. Slowly but assuredly then, with no health issues, and driven by the changes in blood tonicity, participants likely regained their lost water within the next 12–24 h, just in time for another training session, as previously demonstrated by the authors themselves [10] and others [11]. 

In an attempt to contextualize their findings into a real-world context, Adams et al. [1] provided an example and wrote that “…allowing participants to consume fluids during the first 10 min of a post-exercise recovery period, may mimic what could occur in a sport. For example, sports such as soccer and rugby, require athletes to perform continuous exercise, with the elite levels of these sports preventing the number of substitutions permitted; this could create a scenario in which athletes enter the half-time portion of a competition (typically 10–15 min in length) hypohydrated…that may result in marked performance decrement.” Based on this example, participants of the current study would then have entered the second half of the game with a body mass loss of 1.6% when only allowed to drink ad libitum following the dehydrating exercise, which should not be expected to impede performance, based on the actual recommendations [12,13]. Of course, the lack of access to fluid during the second half would lead to a hypohydrated level of >2% body mass. However, in team sports, a recent systematic review has concluded that hypohydration of 3–4% is required to impair performance, a level which, however, is not routinely achieved by these athletes under real-life exercise circumstances [14]. 

In their conclusion, Adams et al. [1] recommend that “…individuals may benefit from knowing their fluid needs and that fluid replacement should be individualized based on fluid losses and subsequent fluid need.” In their 2007 position statement on fluid replacement [13], the American College of Sports Medicine indicated that “Individuals needing rapid and complete recovery from excessive dehydration can drink ~1.5 L of fluid for each kilogram of body weight lost.” According to this recommendation, and within the context of Adams et al.’s [1] example, it follows that participants of their study would have had to consume a whopping 3.5 L of fluid within the half-time period to restore fluid balance. Or, alternatively, taking Adams et al.’s [1] recommendation at face value, it is rather 4.6 L of water that athletes should have consumed, of which one half would serve to replace fluid losses and the other to compensate for the upcoming sweat losses of the second half of the game. Independent of the strategy used, it is highly probable that such amounts of fluid consumed within this short period of time could lead to subsequent stomach bloating and pain, nausea, vomiting, an urge to urinate, or discomfort which, in addition to being detrimental for health, may lead to performance impairment [15,16] or a need to exit from the game field. 

As such, under a scenario similar to that studied by Adams et al. [1], as well as any other where post-exercise body mass loss is important and rehydration needs to be completed rapidly prior to another exercise bout, I propose that following an ad libitum, not a programmed fluid replacement strategy is likely to be the optimal option, at least from a health and performance perspective. Further research looking at the relationship between post-exercise fluid replacement strategies and exercise performance is needed.
